# Class Address

**Published:** 1875-04

**Authors:** James Murphy King


### ARTICLE II.

### *Class Address.*

BY JAMES MURPHY KING, D. D. S.

Delivered at the Thirty-Fifth Annual Commencement of the Baltimore College of Dental Surgery.

*Ladies and Gentlemen :*

There are points in the circle of life—eventful days and moments in which are crowded the joy which shall compensate for years of weary toil and patient endeavor, which have gone before, or may come hereafter.

There are oases in the desert, beneath whose cool palm tree Eve may rest and drink the waters sparkling and purer while by memory's light we retrace the sands over which our weary feet have toiled through days of burning heat; or linger near the solitary tree which has sheltered our head by night.

There are hill-tops in the march of life from whose summit we may sometimes look into the distance which stretches out before us, bright as a poet's dream, fair as October skies.

In the brief hemisphere bounded by the horizon of time, such moments come not often.

It is only after many burning marches that we may sit by the murmuring waters and feel the breath of the palm. It is only after years of toil that we may reach the summit and survey the enchanting scene beyond.

Only he who dares the hero's march may win—only he who has the courage to brave the thorn and rugged rock and dark ravine, may pluck the laurel from the mountain brow.

As we stand to-night in the green of the oases, as we gaze from the mountain brow into the vista which lies before, as a class, we greet you; as a class who have just received the prize for which we have toiled, we thank you for your presence here to night; and in sundering the ties which bind us to each other, and to a Faculty, to each of us endeared, we rejoice that to you no such as word "farewell" must be uttered.

To you we dedicate our lives and our professions—to the good of humanity we consecrate our time and our talents.

I need not to-night review the past of Dentistry—I need not tell you the successive steps by which it has emerged from the dreary night of ignorance and sloth—I need not tell you of the heights to which it has attained—you know it all.

From this stage in days gone by, you have listened to words "which fell like wintry snow" from tongues born to speak, and the perfume of beauteous flowers, and the bright smiles of more beauteous ladies have made known the feelings of your hearts. You know it all. Here in your midst rises like a beacon upon a rocky shore, the oldest College of Dental Surgery in the world.

You have known it from its birth, you have known its childhood and you know its manhood prime. You know

*where* its graduates are to-day,—men from every quarter of the globe, and filling chairs in almost every dental college in the world. You know *who* they are—men of the highest order of education and qualification, men who are competent to elevate and enable whatever their hands find to do.

No, I would rather glance down the vista of the yet to come—and point you to the good which remains to be done and the many strong arms and willing hearts who stand ready to perform it.

And now, woman, with her practised eye and cunning hand is beginning to realize that she has a mission to perform,—that the gates of Dentistry lie open to her, that she may by her presence elevate and honor it, and teach her sisterhood that woman, as well as man, may aspire to something higher and more noble than the relating of the latest gossip, or the discussion of the latest fashion. That aside from her one great and glorious mission on earth, as the companion and helpmeet of man, to bless his pathway, comfort his sorrow, and lead his footsteps heavenward—to mould the mind of infancy, filling it with pure desires, noble aspirations and beautiful hopes; she may in a different sphere glorify her God and benefit her race—administering to the suffering and needy, and as a sister of mercy and disciple of Christ, add her hand to the few but noble hands which are striving to lift the burden from off humanity, the burden of suffering, and sin, and woe, and bring back to earth lost Eden's bloom.

Once more kind friends we tender you our most grateful thanks for your presence here to night, for the kind regard as manifested during our sojourn in your most pleasant city, and for the approving smiles and flowery tributes with which you welcome us on this our Thirty-fifth Anniversary Night.

Gentlemen of the Faculty; the time has come in which must be severed those familiar and pleasing relations of preceptor and student, which have existed during so many months, and we now upon the eve of going forth into the bivance of life, would recall as we shall do many times in the future, the sympathy and courtesy which has ever marked your conduct toward us, and the faithful manner in which you have performed the duties to which Providence has called you; guiding us step by step up the steps of science, taking us as we came to you, raw recruits, and sending us forth to-night thoroughly drilled and fully equipped for our life work.

Gentlemen, a word, a deed, an influence is deathless,—overleaping the shore of time, it is born on the billows of eternity's ocean beyond the judgment day, and we may meet it again somewhere in the infinite future. The influence you exert, the work you do will live forever.

We have sat at your feet in these earthly halls as patient learners; we have looked with you to the rewards of the future; may we one day meet again in the Eternal University, and together sit as students at the feet of the All-wise Teacher. Gentlemen of the Faculty— Farewell.

*Fellow Students.*—Before I close it becomes my pleasant duty to speak a few words relative to the responsibilities and imperative duties that are resting upon you as dental practitioners.

As strangers, we meet at this fountain of instruction for one common purpose, to lay the foundation of that knowledge upon which our future success to a great extent depends. As students, we have been exceedingly fortunate; we have sought and enjoyed the instructions of the oldest Dental College in the world. We have been assisted by the long experience and acknowledged talent of a diligent Faculty, who are efficient in almost every branch of medicine and general surgery, as well as what constitutes the peculiar and distinguishing characteristics of our own special art and science. Our facilities and multiplied appliances for instruction have been full and complete.

Our course of instruction and discipline has been rigid and comprehensive. But complete and thorough as may have been our dental preparations, we have only entered upon our works. We have only laid the foundation upon which to build the monument of our future character and reputations. We have only learned the alphabet of that language, which by assiduous study and diligent cultivation, will disclose to us the beauties, solve the problems, and unlock the hidden treasures of that excellence for which I would bid you strive.

By assiduous study, and manipulative dexterity, we have at last reached all the requirements of graduation as Doctors of Dental Surgery; and we are here to-night to receive from our *alma mater* the reward of our diligence, and to be admitted as worthy members of that special branch of the great art and science of medicine which we have chosen as our profession. And shall our labors stop here? Shall this close our students life? Shall we lay aside our books and conclude that our work is done, that we have nothing more to learn; that we have mastered all the requirements and compassed all the resources of our profession? I trust that there is not one on this stage to-night, who is so dead to enthusiasm, so utterly destitute of ambition as to be satisfied with his present minor attainments.

In severing our connection with this institution, let me admonish you with all earnestness never to let your interest and diligence abate, or your efforts and energies relax, while there remains a single theoretical problem unsolved, or an important practical question undecided. Your diligence and energies instead of abating, should be increased; your research should be more earnest, and assume a more expansive range. You should lend a helping hand in exploring the yet untrodden fields, and solving the yet complex and abstract problems of Dental Science.

As we go forth into the mystic future to meet life's duties, it should be our ever animating spirit and controling power to multiply those resources and augment that knowledge which we have already acquired, and thus "to fulfill the hopes and redeem the trust so confidently reposed in us by our friends and instructors. If we ever attain professional excellence, we must never cease to be students. The imperative is upon us all to labor if we shall ever approximate the "*Ultima Thule*" of our profession—"*Labor omnia vincit.*"

We should consider the passport which we have to-night so joyfully received from the hands of our Dean, not only as a reward of work performed, but rather as an evidence that we are worthy to be admitted into a more useful and extensive field of labor; the diligent cultivation of which will yield to us the highest honors and brightest rewards the world can offer to genius and talent.

Dentistry has but lately arisen to that high status which it now occupies among the learned, artistic and scientific professions. It has but lately received that consideration and esteem which it now so justly merits as a legitimate and honorable specialty of the science of medicine. This being the case, it is imperative upon each one of us to assist in maintaining its high position and to give to it additional reputation. Let us see to it that we not only keep abreast with the progress of the times, but that we leave our footprints on the domain of science.

Gentlemen of the graduating class, a few words more, and I must say to you that word so often uttered on earth sometimes coldly or carelessly, sometimes with beating heart, and eyelids moist—sometimes with bursting sobs—"farewell." The echo of that farewell will ere long die upon the air forever, but not so upon the heart; there it will live while memory lasts, and whenever that word is spoken in the future, it will recall this parting night; it will recall the hours in which we have toiled together here, and our pleasant converse by the way.

We go, but we go to life and to duty. We go to an unknown future, but to each of us it may be a useful future, filled with earnestness and toil and hope of consecration to our work to humanity and to God. And then when life's brief day is rounded with a sleep,. we may sink to rest as calmly as a summer's sun.

There is nothing that can soften a dying bed like a well spent life, nothing that can make the rest of heaven so blest as a life of toil and trial; nothing that can make the songs of glory so sweet as the wail and moan of earth: nothing that can make the everlasting hills so bright with the sunlight of eternity as the darkness and gloom of time.

Gentlemen of the Graduating Class—Farewell.

